# The Peripheral Immune System and Amyotrophic Lateral Sclerosis

**DOI:** 10.3389/fneur.2020.00279

**Published:** 2020-04-21

**Authors:** Pamela A. McCombe, John D. Lee, Trent M. Woodruff, Robert D. Henderson

**Affiliations:** ^1^Centre for Clinical Research, The University of Queensland, Brisbane, QLD, Australia; ^2^Wesley Medical Research, The Wesley Hospital, Brisbane, QLD, Australia; ^3^School of Biomedical Sciences, The University of Queensland, Brisbane, QLD, Australia; ^4^Royal Brisbane and Women's Hospital, Brisbane, QLD, Australia

**Keywords:** amyotrophic lateral sclerosis (ALS), T lymphocytes, monocyte, cytokine, inflammation, immunity

## Abstract

Amyotrophic lateral sclerosis (ALS) is a severe neurodegenerative disease that is defined by loss of upper and lower motor neurons, associated with accumulation of protein aggregates in cells. There is also pathology in extra-motor areas of the brain, Possible causes of cell death include failure to deal with the aggregated proteins, glutamate toxicity and mitochondrial failure. ALS also involves abnormalities of metabolism and the immune system, including neuroinflammation in the brain and spinal cord. Strikingly, there are also abnormalities of the peripheral immune system, with alterations of T lymphocytes, monocytes, complement and cytokines in the peripheral blood of patients with ALS. The precise contribution of the peripheral immune system in ALS pathogenesis is an active area of research. Although some trials of immunomodulatory agents have been negative, there is strong preclinical evidence of benefit from immune modulation and further trials are currently underway. Here, we review the emerging evidence implicating peripheral immune alterations contributing to ALS, and their potential as future therapeutic targets for clinical intervention.

## Introduction

Amyotrophic lateral sclerosis (ALS) is a fatal neurodegenerative disease, defined by the presence of muscle weakness and the progressive death of upper and lower motor neurons (1). ALS leads to respiratory failure with the length of survival being predicted by respiratory muscle weakness (2). However, ALS is more than just a motor neurone disease. ALS also has extra-motor features, including cognitive and behavioral disturbance (3–5). ALS is markedly heterogeneous in clinical features, such as site of onset of weakness and rate of progression (6, 7), and is more common in men than in women (8).

ALS can be sporadic (SALS) or familial (FALS), although the distinction can be difficult to assign (9). Genetic susceptibility (10, 11) and environmental exposure (12) contribute to the pathogenesis of ALS, possibly through a multi-stage process (13, 14). Causative genes exist in patients with FALS, and mutations in these genes occur in some patients with SALS (15). Calculations suggest that 61% of the variance in risk of developing ALS is due to genetic factors (16), which means that ~40% of the variance in risk is due to non-genetic factors, which could include environmental exposures. The pathological features of ALS include aggregation of insoluble protein within cells (17), but the type of protein aggregate varies among patients. It has been thought that the majority of patients have accumulation of tar DNA binding protein 43 (TDP-43), (as well as others), with a small group of patients having accumulations of superoxide dismutase 1 (SOD1) (18–20). However, recent evidence suggests that SOD1 may aggregate in the spinal cord in a majority of ALS patients (21, 22). The genes that cause ALS usually encode for proteins or polypeptides that accumulate within cells or are involved in the metabolism of protein aggregates (19, 23). There is evidence that some of the aggregated proteins can transfer from cell to cell in a prion-like fashion (24, 25) which could explain the characteristic spread of weakness from the site of onset to other regions.

A number of possible pathways of disease have been described, including mitochondrial dysfunction, glutamate excitotoxicity (26, 27), problems with autophagy (28) and altered RNA metabolism (29). Furthermore, the death of motor neurons can be “non-cell autonomous,” meaning that other types of cells such as astrocytes, microglia and possibly oligodendrocytes can drive motor neuron death (30, 31). There has been considerable research on the type of cell death that occurs in ALS. It has been previously thought that neuronal cell death in ALS is due to apoptosis (32–35) which is mediated through caspases. Evidence for apoptosis in ALS has been found with TUNEL staining of human tissues (36) and with measurements of bcl-2 (37). Others found increased p53 in ALS (38). In ALS there is also evidence of caspase activation (35). However, more recently there has been a suggestion that necroptosis, an inflammatory form of cell death which is caspase independent and involves RIP kinase activation, is a common form of cell death in neurodegenerative disease (39). Necroptosis is the mechanism of cell death from glutamate toxicity (40), which is one of the most important mechanisms proposed for the pathogenesis of ALS. There is evidence that necroptosis occurs in a cell culture model of ALS (41). Mutations in optineurin, a rare genetic cause of ALS, allow the activation of RIP kinases to promote necroptosis (42). More recently still, ferroptosis, an oxidative form of cell death (43), has been reported to occur in ALS (44).

The death of motor neurons, possibly stimulated by the pathways described above, and occurring through one of the types of cell death described above, is the cardinal feature of ALS. However, the pathology of ALS in the brain and spinal cord also involves more than death of motor neurons, with evidence of involvement of the immune system (45). There is neuroinflammation with microglial activation and a modest level of T lymphocyte infiltration (46–48). In ALS patients, microglial activation is visible with PET imaging suggestive of an ongoing neuroinflammatory process (49). Such inflammatory pathology could be a reaction to cellular damage. Once established, such inflammation could aggravate disease. However, it must be also noted that the immune system can also be protective, particularly after injury (50, 51). Thus, the role of the immune system in pathogenesis could be either harmful or helpful, and work is required to delineate the precise role of each immune pathway to ALS pathology.

There is also evidence of abnormality of the peripheral immune system in ALS, and this is the topic of the present review. As with inflammation in the CNS, peripheral immune activation could be a reaction to tissue damage, but once established, could exacerbate disease. This review will focus on describing the abnormalities of circulating blood cells, different immune system proteins, and their key inflammatory mediators, cytokines. These are summarized in Tables 1, 2. To consider whether the immune abnormalities contribute to disease pathogenesis, we list some evidence that these abnormalities are correlated with human disease or are pathogenic in animal models of ALS. If immune abnormalities contribute to pathogenesis, then modification of the immune response could be beneficial to patients, so we also highlight the results of forthcoming and completed clinical trials of immune interventions in ALS.

**Table 1:** Changes in peripheral blood cells in ALS.

**Cell**	**Change in ALS**	**Evidence of disease association**	**Reference(s)**
Total leukocytes	Increased	Level correlates with rapidity of progression	(52)
Granulocytes/neutrophils	Increased granulocytes, increased neutrophils, increased ratio of neutrophils to monocytes	Treatment with mastinib reduces axonal degeneration in animal model	(53–56)
CD4^+^T cells	Conflicting reports, most suggest an increase	CD4^+^ T cells are protective in animal model	(54, 57–60)
CD8^+^ T cells	Conflicting reports	Cytotoxic cell cause death of motor neurones in animal model	(54, 57, 59, 61, 62)
NK T cells	Increased	Reduction in numbers led to prolonged survival in animal model	(61, 63)
Treg cells	Reduced and dysfunctional	Inverse correlation with rate of progression	(57, 61, 64–66)
CD14^+^ monocytes	Variable reports of numbers, but evidence of activation, increased ratio of classical to non-classical monocytes	Monocyte activation correlates with disease progression	(52, 57, 58, 67–69)

**Table 2:** Changes in peripheral blood proteins in ALS.

**Protein**	**Changes in ALS**	**Evidence for role in pathogenesis**	**References**
IgG	Increased	Passive transfer leads to motor neurone degeneration	(70–72)
Complement	Increased complement in ALS	Lack of C5a is protective in animal model	(59, 73–75)
Tumor necrosis factor	Increased	Mixed effects on motor neuron survival, depending on receptor	(76–82)
Interleukin 1β	Increased	Blocking IL-1 led to prolonged survival in animal model of ALS	(77, 79, 83)
Interleukin 33	Reduced	Treatment with IL33 reduced disease in animal model of ALS	(84, 85)
Interleukin 6	Increased	Genetic variation of IL-6 receptor influences the severity of ALS. However, IL6 deficiency has no effect of animal model	(79, 86–90)
Interleukin 17	Increased	Unknown- but usually pro-inflammatory	(91)
C reactive protein	Increased	Unlikely—this is evidence of inflammation	(89, 92–94)

## Abnormalities of Peripheral Blood Cells

### Total Leukocyte Count/Granulocytes

Several studies have provided evidence of immune activation in the peripheral blood in ALS. The total leukocyte count is elevated in patients with ALS, and correlates with progression of disease (52). The ratio of neutrophils to monocytes was also shown to be increased (53), as was the total number of granulocytes (54). A micro-array study further confirmed evidence for mild neutrophilia in ALS patients (55). In the SOD1^G93A^ transgenic mouse ALS model, circulating neutrophils are increased (73), and neutrophils and mast cells are present along peripheral motor axons, with masitinib treatment leading to reduction of axonal damage (56). This suggests these cells are harmful and contribute to disease progression.

### Lymphocytes

#### CD4^+^ T Cells

Some studies demonstrate increased levels of CD4^+^ helper T lymphocytes in patients with ALS (54, 57, 58), but others have found reduced numbers of these cells (59). It is possible that this variation is related to the variation in immune responsiveness of individuals. CD4^+^ T cells in the CNS are thought to be neuroprotective in an animal model of ALS (60), and a lack of CD4^+^ T cell mediated neuroprotection could be detrimental, in patients with reduced numbers. This protection is mediated through Treg cells that are discussed below.

#### CD8^+^ T Cells

There are reports of reduced levels of CD8^+^ cytotoxic T lymphocytes in ALS (57), reports of increased levels of CD8^+^ cytotoxic T lymphocytes (54, 61), and reports of no alterations in these cells (59). Once again this could be related in part to individual variability. In the SOD1^G93A^ mouse ALS model, cytotoxic lymphocytes cause death of motor neurons (62), so increased numbers could be detrimental.

#### NKT Cells

NK T cells recognize lipid antigens through CD1,and secrete an array of cytokines (95). A study in people with ALS found increased levels of natural killer T (NKT) cells (61). In the SOD1^G93A^ mouse model, there are also increased NKT cells, especially in the liver (63); furthermore treatment that reduced the numbers of peripheral NKT cells led to prolongation of life-span, suggesting that these cells are harmful in ALS.

#### Th 17 Cells

The co-stimulatory pathway activated through CD40 ligand is upregulated in some human subjects with ALS (96), and there is thought to be a particular activation of Th-17 T lymphocytes (97). Th17 lymphocytes are pro-inflammatory and thought to be harmful, but can exhibit plasticity and change to other less harmful functions (98).

#### Treg Cells

Much work has focused on regulatory T cells (Tregs) in ALS (99). There are reduced levels of Tregs in ALS patients (57, 61, 64), and these cells are also found to be dysfunctional (65). The level of Tregs correlates inversely with progression of disease (64). Another study also found that there was an inverse correlation between Treg numbers and the rate of disease progression (66). In a human trial, three patients were given autologous expanded Tregs (100), which showed a possible reduction in the rates of disease progression during infusion periods. A trial has been commenced to determine whether rapamycin, which increases levels of Tregs through the mToR pathway, can lead to increased levels of Tregs in ALS (101).

In SOD1^G93A^ mice, there is evidence of dysfunction of Tregs and transfer of wild-type Tregs delays onset of disease (102). Another study in SOD1 transgenic mice showed that transfer of Tregs slowed disease progression (66). These studies are a promising area of research because of the suggestion that Tregs are able to control or reduce disease activity, but clearly requires larger, controlled and blinded human studies to validate their therapeutic potential.

### NK Cells

NK cells are cells of the innate immune system, that mediate cytotoxicity. There is an increase in NK cells in patients with ALS compared to controls (52, 54). NK cells are found in the CNS of SOD1 G93A mutant mice where they are thought to be harmful. Thus, NK cells could possibly be pathogenic and a trial of anti-NK therapy has been proposed in ALS (http://grantome.com/grant/NIH/R21-NS102960-01A1).

### Monocytes

#### Monocyte Classification

With measurements of expression of CD14 (the lipopolysaccharide receptor) and CD16 (the FcγIII receptor), monocytes can be separated into three groups; these are classical (CD14^++^ CD16^−^), intermediate (CD14^++^CD16^+^) and non-classical (CD14^+^CD16^++^) (103, 104). HLA DR is expressed in CD16^+^ monocytes, while CD14^+^ monocytes reduce HLADR expression when activated (105). Other markers can also be used to distinguish monocyte subsets (106).

#### Monocyte Numbers and Proportions

There is a report of a mild increase in CD14^+^ monocyte numbers in ALS (52). However, another study reported reduced levels of CD14^+^ cells in the early stage of disease (57). There is also a report that there is no difference on the numbers of CD14^+^ monocytes between patients and controls (58). In addition, it has been reported there is a reduction in CD16^−^ monocytes in ALS (53). These variations could be explained by differences in methodology and the lack of clear demarcation between these monocyte populations in flow cytometry gating strategies. There are also reports of alterations in the proportions of monocytes in ALS, with an increase in the ratio of classical to non-classical monocytes (67, 107).

In addition to population shifts, there have been reports of alterations in monocyte activation in ALS. CD14^+^CD16^−^ classical monocytes in ALS show an inflammatory microRNA profile (68). Another study reported increased production of neurotoxic cytokines by monocytes from twins with ALS compared to the unaffected twin (108). Increased peripheral monocyte expression of inflammatory genes correlates with disease progression (69). Another study reported expression of activation markers on monocytes but reduced expression of HLA-DR (57). In another study, patients with ALS could be separated into groups, with one group showing increased HLA-DR expression on monocytes (54). Another study found that there was increased expression of HLA-DR on CD14^+^ monocytes in ALS and this correlated with the rate of disease progression (58). Another study used exosomes to activate monocytes, and found that monocytes from ALS patients were less responsive than those from healthy individuals (109). ALS monocytes are less responsive to purinergic stimulation than those from controls (110).

As outlined above, the pathology of ALS is characterized by the accumulation of aggregates of proteins in neurons. There is now evidence of abnormal accumulation/location of these proteins in monocytes. For example, altered location of TDP43 in monocytes of patients with genetic mutations in TARDBP, the gene encoding TDP-43 has been demonstrated (111). There is also a report that C9orf72 is expressed in myeloid cells and that expression in monocytes increases after activation (112). Ablation of the mouse homologue of C9orf72 led to macrophage dysfunction and microglial activation (113).

There is evidence that peripheral monocytes enter the CNS in ALS (107), although this is controversial. In SOD1^G93A^ mice, numbers of inflammatory monocytes correlated with disease progression (114). Activated macrophages are found around degenerating nerve (115) and at the neuro-muscular junction in mouse models of ALS (116). These experimental studies suggest that a shift toward activated monocytes in ALS could contribute to ALS progression through secretion of inflammatory and potentially neurotoxic mediators. Further research is needed to precisely define the role of the monocyte in ALS.

## Abnormalities of Immune Proteins in Peripheral Blood

### Immunoglobulin Levels

Some of the first studies of the role of the immune system in ALS were concerned with the presence of antibodies in the blood of subject with ALS, particularly reports of antibodies to voltage gated calcium channels (117, 118). In addition, there have been studies of non-specific changes in antibodies, as a recent study has shown an increase in IgG levels in subjects with ALS compared to controls (70). In mice, an experimental study showed that prolonged intra-peritoneal injection of immunoglobulin from human subjects with ALS led to loss of spinal motor neurons and loss of muscle strength (71). An earlier study showed that passive transfer of purified immunoglobulin from ALS patients led to motor neuron degeneration and accumulation of calcium containing organelles (72).

### Complement System

There is clear evidence of activation of innate immune complement system in human subjects with ALS, with raised C5a levels and increased expression of C5a on human leukocytes (74). A two dimensional gel electrophoresis was used to study serum proteins in ALS subjects and found that components of complement C3 were increased compared to controls (75) and another study using nephelometry showed increased levels of complement C3 in the blood of ALS patients (59). Animal studies also indicate a role for terminal complement activation in motor neuron degeneration. In SOD1 and TDP43 animal models of ALS there is evidence of complement activation (119, 120), and genetic deficiency or pharmacological inhibition of the C5a receptor, C5aR1, is protective in rodent SOD1^G93A^ models (73, 121–123). A comprehensive review of the involvement of complement in ALS has recently been written (124).

### Cytokines

#### Tumor Necrosis Factor (TNF)

There are increased levels of TNF and soluble TNF receptor in the blood of patients with ALS (76–78). A meta-analysis found that TNF levels were significantly increased in ALS (79). RNA-seq analysis has identified TNF as a contributor to inflammation in the spinal cord of ALS patients (125). It is unknown whether this inflammation is harmful or beneficial. It has been suggested that TNF is harmful and that reduction would be beneficial (80). On the other hand, TNF stimulates a survival pathway in motor neurons and could be beneficial (81, 82).

In SOD1 mutant mice, signaling through the TNF receptor 2 lead to motor neurone death (126), whereas signaling through TNF receptor 1 was harmful (127). The recent suggestions that TNF inhibitors could be a risk factor for ALS (128) could indicate that TNF is beneficial in some way.

#### Interleukin 1 (IL-1)

Interleukin 1 exists as a family of proteins (129). One study found that interleukin 1 β (IL-1β) was undetectable in ALS patients (130) but other studies have found increased levels (77). A meta-analysis found that IL-1β was significantly increased in ALS (79). Pathways involving IL-1 are thought to be involved in ALS pathogenesis as shown in SOD1 and TDP-43 animal models (83, 131, 132). A proteomic study of plasma from ALS patients showed activation of pathways associated with inflammation and activation of two networks centered on NF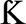 B and IL-1 (133). In animal studies, blocking IL-1 led to prolonged survival (83). In humans, there has been a pilot study that showed that blocking IL-1 with Anakinra was safe in ALS, although there was no prolongation of survival (134).

#### Interleukin 33 (IL-33)

IL-33, a cytokine related to IL-1, has a role both in inflammation, and in metabolism (135, 136). IL-33 binds to receptor, ST2. Levels of IL-33 are reduced in ALS, and levels of soluble ST2 are increased in ALS (84). In a study in SOD1^G93A^ transgenic mice, IL-33 treatment ameliorated disease (85), suggesting this is a key downstream mediator of ALS progression.

#### Interleukin 6 (IL-6)

IL-6 is considered to be a pro-inflammatory cytokine, and is part of an acute phase response, however, it also has some documented anti-inflammatory effects. Plasma levels of IL-6 are increased in ALS (79, 86, 87), and this was supported by a meta-analysis (79). One study suggested that this was a response to hypoxia rather than to the disease itself (137) (see below). IL-6 has been suggested to have a role in endothelial damage in ALS (138). Genetic variation in the IL-6 receptor has been shown to modify the severity of ALS (88). Treatment with the IL-6 blocking antibody toclizumab reduced levels of IL-6 and other cytokines in cells from some ALS patients (89); this study did not look for effects on clinical signs. In SOD1 mutant mice, IL-6 deficiency did not affect the severity of disease (90).

#### Interleukin 17 (IL-17)

IL-17 is a pro-inflammatory cytokine that also responds to stress (139, 140). Increased levels of IL-17 are reported in the serum of subjects with ALS (91, 141), but to date IL-17 has not been explored clinically as a therapeutic target.

#### Interleukin 13 (IL-13)

IL-13 regulates T lymphocytes and has been implicated in autoimmune disease (142). IL-13 levels are elevated in the blood of patients with ALS (77). IL-13 producing T lymphocytes have been found in the blood of subjects with ALS and correlate with the rate of disease progression (91, 143).

#### Interleukin 18 (IL-18)

IL-18 is another member of the IL-1 family of cytokines and stimulates many lymphoid cells (144). Although levels of IL-18 are increased in patients with ALS (130), there is no information about relation of IL-18 to disease activity to date.

### Chemokines

Chemokines are small proteins that are involved in chemotaxis and activation of granulocytes and lymphocytes. In the CNS, chemokines also have a role in signaling between cells (145). The expression of MCP-1 receptor (CCR2) is reduced on circulating monocytes in ALS (146). Another study showed significantly increased expression of CXCR3, CXCR4, CCL2, and CCL5 on T lymphocytes in ALS patients compared to healthy controls (147). There are higher levels of the chemokine MCP-1 in patients with a shorter diagnostic delay, which is a marker of more severe rapidly progressing disease (148).

### Other Evidence of Systemic Inflammation

There is also evidence of increased levels of C reactive protein and erythrocyte sedimentation rate (ESR) in subjects with ALS compared to controls, and evidence that levels correlate with the levels of disability as measured by the ALS functional rating scale (89, 92–94). Levels of lipopolysaccharide are elevated in patients with ALS, (149), as have levels of nitric oxide, suggesting systemic inflammation (78).

### Evidence of Hypoxia

In ALS, there is evidence of hypoxia in neurons, and this is thought to contribute to pathogenesis. This can be seen as increased levels of hypoxia inducible factor−1α (150). There is also thought to be dysregulation of the pathways that protect from hypoxia (151, 152). In the peripheral blood monocytes of ALS patients there is also dysregulation of hypoxia pathways (153). A gene expression study found evidence of hypoxia related genes in peripheral blood of ALS patients (55). In an animal model of ALS, hypoxia aggravates the loss of motor neurons (154). The significance of these findings is presently unclear, but this is further evidence of peripheral immune changes in ALS.

### NF-κ B Pathways

Nuclear factor κB (NF-κ B) is a protein complex that regulates the transcription of DNA. Evidence that NF-κ B is important in ALS comes from studies showing genetic abnormalities in optineurin (155). Analysis of cell transfection showed that the nonsense and missense mutations of OPTN abolished the inhibition of activation of NF-κ B. The authors proposed that NF-κ B is the final common pathway in ALS pathogenesis, and that inhibitors of NF-κ B could be used to treat ALS. Further, in animal studies it has also been found that the NF-κ B p65 subunit is a binding partner for TDP-43 and that dysregulation of TDP-43 leads to activation of NF-κ B (156). NF-κ B is expressed in astrocytes (157), and in activated microglia in ALS spinal cord (158).

Other evidence of a possible role of NF-κ B in ALS comes from a role for hypoxia in ALS. (153, 159) (see above). NF-κ B is activated during acute hypoxia and acts to up-regulate inflammatory factors such as IL-6, cyclo-oxygenase (COX 2), TNF-α, and prostaglandin E-2 (PGE-2) (159). Reactive oxygen species lead to induction of NF-κ B. This is mainly in lymphoid cells but also in neurones. It has been suggested that NF-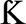 B is a transcription factor controlled by hypoxia and may contribute to neurological disorders (160).

In neurodegenerative disease it is thought that NF-κ B can augment cell death (161). There is some evidence about the role of NF-κ B from animal models of ALS. In SOD1^G93A^ mutant mice, treatment with a PPAR inhibitor led to clinical improvement and reduced expression of iNOS and NF-κ B reactivity (162). Phenylbutyrate induced NF-κ B translocation to the nucleus in ALS mice, and this led to reduced motor neuron death (163). Intrathecal injection of an adenovirus containing insulin like growth factor led to slowing of disease through inhibition of NF-κ B in an animal model of ALS (164). However, inhibition of NF-κ B in astrocytes did not reduce disease in ALS mice (165).

The above-mentioned studies focus predominantly on the role of NF-κ B within the CNS in ALS. Little is known about NF-κ B in the peripheral immune system in ALS, but given the central role of NF-κ B in the biology of the immune system (166), this warrants further study.

### Immunometabolic Changes

There is considerable interaction between the immune system and metabolic pathways, which is a rapidly growing research field being known as “immunometabolism” (167, 168). For survival, metabolism and the immune system need to be linked, because there needs to be a mechanism for balancing the energy needed for basal and defensive processes (169). In ALS, there is evidence of alterations in metabolism (170). There are reports of alteration in the levels of metabolic proteins such as adipokines. This includes IL-6 but also other proteins such as leptin and adiponectin (86, 171). A proteomic study found dysregulation of pathways involved in lipid metabolism (133). In particular, there was dysregulation of the Liver X receptor/Retinoid X receptor (LXR/RXR) and the Farnesoid X receptor/Retinoid X receptor (FXR/RXR) pathways that are at the intersection of immunology and metabolism.

### Evidence From Completed Clinical Trials

It is attractive to consider that modulation of the immune response will be a useful therapy in ALS. If neuroinflammation enhances disease activity, then control of neuroinflammation should be helpful (172), possibly by enhancing the protective immunity (173).

Overall, clinical trials of new disease-modifying therapies in ALS have been disappointing (174). Several of these trials have used medications that act on the peripheral immune system. Total body irradiation and stem cell therapy were of no benefit in ALS (175). Earlier attempts at immune therapy included treatment with intravenous immunoglobulin, (176), with cyclophosphamide, (177) and with azathioprine and prednisone which were also of no benefit (178). Glatiramer acetate, a synthetic polypeptide with immune effects that is used in multiple sclerosis, further demonstrated no benefit in ALS (179).

Minocycline, an anti-inflammatory agent, also failed in a trial in ALS, and in fact, patients on this treatment had a worse outcome (180). Celecoxib, another anti-inflammatory agent, also failed its clinical end-point (181). Sodium chlorite (NP001) which was proposed to deactivate macrophages, was also more recently shown to be unsuccessful (182, 183).

Masitinib, a tyrosine kinase inhibitor that targets mast cells, microglia, and macrophages despite showing positive results in SOD1 transgenic mice (184), failed in its phase II study in humans (185). Finally, a trial of granulocyte colony stimulating factor led to a decrease in levels of MCP-1 and IL-17 in subjects with ALS (186).

## Is There an inflammatory Subgroup?

One of the challenging features of ALS is its heterogeneity- of clinical features, of rate of progression and also in the underlying pathological aggregation of proteins. This heterogeneity could indicate that the pathogenesis of disease varies among patients, and there could be sub- groups of patients in whom immune processes are more or less important.

A study of gene expression indicated that patients can be grouped into patients with higher expression of IL-6R and myeloid lineage-specific genes, and patients with higher expression of IL-23A and lymphoid-specific genes (55). The results from a clinical trial of Toclizumab also led the authors to note that Toclizumab reduced IL-6 and other cytokines in cells from some ALS patients (i.e., an “inflammatory group”) but not others (89).

## Interaction Among the Nervous System, the Immune System and the Gut Microbiota

The gut microbiota has been increasingly recognized as playing an important role in human health, and has been implicated in neurodegenerative disease including ALS, as we have recently reviewed (187). One of the many functions of the gut microbiota is to regulate the immune system. It is therefore possible that some of the immune abnormalities in ALS are linked to the gut microbiota. However, this field is complex and analysis requires large numbers of subjects so more remains to be discovered regarding this possible interaction.

## Immunogenetics of ALS

If immune genes played a role in the susceptibility to ALS or modified the course of ALS, this would be evidence of involvement of the immune system in disease. In autoimmune diseases, there is an association of disease with HLA loci (188). This is not the case in ALS, except for a possible association with HLA class I antigens (189, 190).

The association with HLA class I antigens could be due to linkage with the haemochromatosis locus (HFE), which is found in the HLA region. Some years ago, an association with the H63D polymorphism was reported (191). More recently, a meta-analysis has discounted this association but instead suggested an association with the C282Y polymorphism (192).

There are numerous polymorphisms that affect the immune system. These have been linked to autoimmune diseases such as multiple sclerosis and type I diabetes (193) but not to ALS (194). However, it would seem likely that genetic variation in immune genes could influence the immune abnormalities described above, For example, polymorphisms in cytokine genes can influence the levels of cytokines such as IL-6, (195) and TNFα (196) and the IL33/ST2 pathway (197).

The field of immunogenetics of ALS would appear to be a fruitful topic for further exploration, and possibly could explain why some patients have a stronger immune response than others, and why some patients show an “inflammatory” phenotype. Immunogenetics, and variation in the immune response to disease could therefore contribute to the known heterogeneity of ALS.

## Conclusion

There is clear evidence of immune activation in some patients with ALS and in animal models of disease. It is possible that there is a subgroup of patients in whom inflammatory pathways are important in pathogenesis. In some cases, the immune abnormalities are correlated with disease severity, but it is not clear whether this is cause or effect. The immune system has both harmful and beneficial effects and there is a need to focus research efforts on enhancing the beneficial effects of protective immunity. Clinical trials so far have been disappointing, but there is still scope for further attempts at immune intervention to ameliorate this disease.

## Author Contributions

PM conceived the idea and wrote the first draft. JL, TW, and RH read and revised the manuscript.

## Conflict of Interest

The authors declare that the research was conducted in the absence of any commercial or financial relationships that could be construed as a potential conflict of interest.
